# Toward Marker-Assisted Selection in Breeding for *Fusarium* Wilt Tropical Race-4 Type Resistant Bananas

**DOI:** 10.3390/jof10120839

**Published:** 2024-12-04

**Authors:** Claudia Fortes Ferreira, Andrew Chen, Elizabeth A. B. Aitken, Rony Swennen, Brigitte Uwimana, Anelita de Jesus Rocha, Julianna Matos da Silva Soares, Andresa Priscila de Souza Ramos, Edson Perito Amorim

**Affiliations:** 1 Embrapa Mandioca e Fruticultura, Rua Embrapa s/n CP 007, Bairro Chapadinha, Cruz das Almas 44380-000, Bahia, Brazil; andresa.ramos@embrapa.br (A.P.d.S.R.); edson.amorim@embrapa.br (E.P.A.); 2 School of Agriculture and Food Sustainability, The University of Queensland, St. Lucia, QLD 4072, Australia; a.chen2@uq.edu.au (A.C.); e.aitken@uq.edu.au (E.A.B.A.); 3 International Institute of Tropical Agriculture, Banana Breeding, Kampala P.O. Box 7878, Uganda; rony.swennen@kuleuven.be (R.S.); b.uwimana@cgiar.org (B.U.); 4 Department of Biosystems, KU Leuven University, Willem De Croylaan 42, bus 2455, 3001 Leuven, Belgium; 5 Department of Biological Sciences, State University of Feira de Santana, Feira de Santana 44036-900, Bahia, Brazil; anelitarocha@gmail.com (A.d.J.R.); juliannamatos91@gmail.com (J.M.d.S.S.)

**Keywords:** breeding, cleaved amplified polymorphic sequences markers, *Fusarium oxysporum* sp. *cubense*, *Musa acuminata* ssp. *malaccensis*

## Abstract

*Fusarium* wilt is a soil borne fungal disease that has devastated banana production in plantations around the world. Most Cavendish-type bananas are susceptible to strains of *Fusarium oxysporum* f. sp. *cubense* (*Foc*) belonging to the Subtropical Race 4 (STR4) and Tropical Race 4 (TR4). The wild banana diploid *Musa acuminata* ssp. *malaccensis* (AA, 2n = 22) carries resistance to *Foc* TR4. A previous study using segregating populations derived from *M. acuminata* ssp. *malaccensis* identified a quantitative trait locus (QTL) (12.9 cM) on the distal part of the long arm of chromosome 3, conferring resistance to both *Foc* TR4 and STR4. An SNP marker, based on the gene Macma4_03_g32560 of the reference genome ‘DH-Pahang’ v4, detected the segregation of resistance to *Foc* STR4 and TR4 at this locus. Using this marker, we assessed putative TR4 resistance sources in 123 accessions from the breeding program in Brazil, which houses one of the largest germplasm collections of *Musa* spp. in the world. The resistance marker allele was detected in a number of accessions, including improved diploids and commercial cultivars. Sequencing further confirmed the identity of the SNP at this locus. Results from the marker screening will assist in developing strategies for pre-breeding *Foc* TR4-resistant bananas. This study represents the first-ever report of marker-assisted screening in a comprehensive collection of banana accessions in South America. Accessions carrying the resistance marker allele will be validated in the field to confirm *Foc* TR4 resistance.

## 1. Introduction

Bananas are among the leading fruits that provide economic sustainability for both small- and large-scale growers, with a worldwide trade value surpassing USD 30 billion per annum [1]. However, banana production has been severely affected by major pests and diseases caused by viruses, bacteria, and fungi. Amongst the fungal diseases affecting bananas, *Fusarium* wilt, caused by *Fusarium oxysporum* f. sp. *cubense* (*Foc*), is the most economically important.

*Fusarium* wilt has been an issue in banana monoculture plantation production systems since the 1900s. However, it is the virulent *Foc* strain known as Tropical Race 4 (TR4) that threatens commercial banana production dominated by Cavendish-type clones, as well as a broad range of other banana varieties [2,3,4]. TR4 was first reported in the regions of southeast Asia and Australia in the 1990s [5]. In recent years, it has made its way into other parts of the world, such as Latin America, reaching the northern region of La Guarija, Colombia, in 2019 [3], the Querecotillo district of Sullana province in the Piura department of Peru in 2021 [2], and more recently, Venezuela [6]. These countries could provide proxies in the potential spread of *Foc* TR4 across the border to neighboring countries. Thus, pre-emptive breeding to contain and stop the spread of the disease is of utmost importance. Latin America and the Caribbean, which is the world’s largest banana exporting region, is thus endangered [7]. Understanding the epidemiology of *Fusarium* wilt in bananas is critical for developing control measures, breeding resistant varieties, and formulating effective quarantine strategies to prevent further spread of *Foc* TR4 in banana-producing regions of the world [8,9]. *Foc* TR4 remains a critical threat to banana plantations worldwide, partly due to the lack of effective control measures and limited sources of *Fusarium* wilt resistance within *Musa* spp. [8,10,11].

Disease symptoms are manifested as vascular discoloration in the corm, while the disease is visually identified on plants displaying leaf yellowing and vascular-induced wilt. *Foc,* a soilborne pathogen, enters the host plant through the roots and moves through the plant’s vascular system to colonize the whole plant. Its colonization and proliferation in the xylem vessels block water and nutrient supplies to the aerial parts of the plant, leading to wilting of leaves and eventual plant death [8,10]. Internally, *Foc*-infected corms and pseudo-stems show a reddish-brown discoloration. So far, there are no effective means to control the spread of this disease. *Foc* can survive in the soil for decades as chlamydospores and cannot be completely eradicated [12]. Therefore, the use of resistant cultivars not only offers the most sustainable and environmentally friendly means of controlling *Foc* but is perhaps one of the few viable options [13]. This highlights the importance of identifying resistance in wild relatives of cultivated bananas and introducing resistance alleles into commercial cultivars to enhance their resilience against diseases [4,9].

Embrapa Mandioca e Fruticultura (Embrapa Cassava and Fruits, https://www.embrapa.br/mandioca-e-fruticultura, accessed on 21 October 2024) conducts research to improve crop productivity and has an active program for the genetic improvement in bananas since 1976 [14]. It maintains one of the largest banana germplasm collections in South America. This collection includes accessions gathered from various regions worldwide, established through comprehensive collecting expeditions. These expeditions were conducted in the banana center of origin in Southeast Asia and further extended to include accessions from Africa, Central America, and South America. This germplasm collection now has over 400 accessions, aimed at preserving genetic diversity and developing new cultivars suited to both local and global agricultural needs.

Molecular markers based on Single Nucleotide Polymorphisms (SNPs) are used successfully in many crops in the identification of a plant’s resistance to diseases [15,16,17,18]. Previous studies show that lines belonging to the wild diploid banana *Musa acuminata* subsp. *malaccensis* (Ma) (AA, 2n = 22) carry resistance to *Foc* TR4 and STR4 strains [8,10,17,18].

Recently, a detailed study of two *Musa acuminata* ssp. *malaccensis* populations segregating for *Foc* TR4 and STR4 resistance was carried out [18]. Marker loci and trait association using 11 SNP-based PCR markers allowed the candidate region conferring *Foc* STR4/TR4 resistance to be delimited to a 12.9 cM genetic interval corresponding to a 959 kb region on chromosome 3 of the ‘DH-Pahang’ reference assembly v4 [18].

Within this region, multiple pattern recognition receptors, such as leucine-rich repeats containing receptor-like protein kinases, cysteine-rich cell wall-associated protein kinases, and other genes related to resistance, were identified. To confirm the segregation of single-gene resistance, an inter-cross between the resistant parent ‘Ma850’ and a susceptible line ‘Ma848’ was generated [18]. An informative SNP marker, 29730, based on a single nucleotide change (T544C) in the first intron of a Nuclear transcription factor Y subunit (Macma4_03_g32560, ‘DH-Pahang’ assembly v4.3) was reported as associated with *Foc* TR4 resistance [18] and allowed the resistance locus to be assessed in a collection of diploid and polyploid banana accessions at Embrapa. The results provide crucial insights into *Foc* TR4 resistance sources within Embrapa’s banana breeding program, supporting the design of a breeding pipeline geared toward the development of *Foc* TR4-resistant bananas and as such, mitigate TR4-caused yield losses once TR4 arrives in Brazil. These findings will undoubtedly contribute to the global initiatives aimed at developing *Foc* TR4-resistant banana varieties.

## 2. Materials and Methods

*M. acuminata*-specific primers for the 29730 marker were developed previously, 29730-A-SNP1-F2 5′-GCAATGAGTACCTCTAAGCA-3′ and 29730-A-SNP1-R2 5′-TAAGTTCTAGTATCAAGTACAA-3′, and used to amplify an A-genome specific product of 795 bp. This product was then digested with BcoDI to produce the bi-allelic forms, an undigested dominant band that is putatively associated with resistance, and digested products of 429 bp and 366 bp, linked to susceptibility [18]. Then, 123 accessions from the Embrapa banana germplasm collection (Table 1) were genotyped with this SNP marker, 29730-A-SNP1-F2 and 29730-A-SNP1-R2.

## 3. Results and Discussion

**Figure 1:**
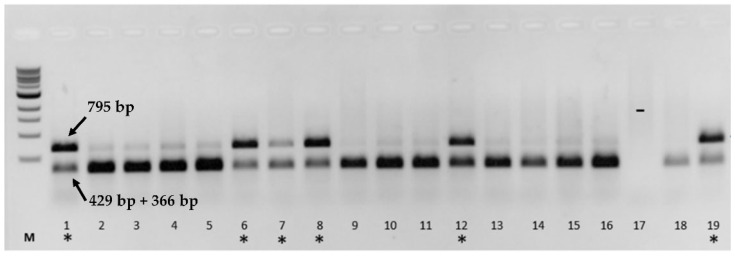
BcoDI digest profile of 19 Embrapa accessions PCR-amplified using the SNP marker 29730-A-SNP1-F2 and 29730-A-SNP1-R2. The 795 bp fragment is putatively associated with resistance. Samples are (1) ‘PA Mysore 2’, (2) ‘Pisang Jari Buaya’, (3) ‘Tuu Gia’, (4) ‘Pisang Nangka’, (5) ‘Tjau Lagada’, (6) ‘Tong Dok Mak’, (7) ‘M-61’, (8) ‘Pisang Mas’, (9) ‘Pisang Cici’, (10) ‘Saney’, (11) ‘Yangambi no.2’, (12) ‘Yangambi Km5’, (13) ‘Highgate’, (14) ‘Pacoua PV03-76’, (15) ‘PA 42-38’, (16) ‘Pa 42-28’, (17) ‘PA 42-19’, (18) ‘M48’, and (19) ‘Ouro’. M = molecular ladder 1 kb (N3232S, New England Biolabs, VIC, Australia), (-): Non-amplified sample. The alternatively cut allele (429 bp and 366 bp) may indicate the presence of the *Foc* TR4-susceptible allele. Accessions heterozygous for the marker locus are predicted for resistance to *Foc* STR4 and *Foc* TR4 given the complete dominance of resistance over susceptibility at this resistance locus. Asterisk = putative *Foc* TR4/STR4-resistant genotypes.

The overall results of the marker-assisted screening for all 123 accessions are summarized in Table 1. A restriction digest was performed using BcoDI on PCR amplicons of all 123 Embrapa accessions. A BcoDI cut site within the PCR amplicon allowed bi-allelic forms to be discriminated on an agarose gel (Figure 1). Accessions displaying a dominant resistant band were subsequently analyzed through Sanger sequencing. The sequencing results confirmed the presence of the informative SNP, as identified in the alignment of chromatograms at the expected position (Figure S1).

The SNP marker was screened in all putative genotypes for resistance. PCR products of selected accessions were Sanger-sequenced in both directions by ACTGene Company, using the AB3500 sequencing platform (ACTGene, Porto Alegre, RS, Brazil). Of the retrieved sequences, these were analyzed using the multiple-sequence alignment software MAFFT v 7.490 [20] in Geneious Prime v 2024.0.7 (Biomatter Pty. Ltd., Auckland, New Zealand). Overall, 14 genotypes, out of 15, showed the presence of the resistance allele (Supplementary Figure S1). Of these, three improved diploids have Malaccensis in their genetic background, namely ‘013019’, ‘CNPMF 0542’, and ‘CNPMF 0731’. Given that the breeding program at Embrapa focuses on a preventive breeding strategy to combat the disease, these improved diploids will play a key role in the development of *Foc* TR4-resistant cultivars aimed at enhancing resistance to *Fusarium* wilt in the field.

Among the 15 accessions previously identified as highly resistant or resistant to *Foc* STR4 [19], 10 were associated with the resistance marker band. These included the improved diploids CNPMF- ‘0496’, ‘0534’, ‘0536’, ‘0542’, ‘0557’, ‘0731’, ‘013019-01’, ‘050012-02’, ‘SH3263’, and ‘SH3362’ (Table 1). However, five accessions, including CNPMF- ‘1323’, ‘0513’, ‘0993’, ‘0998’, ‘001016-01’, and ‘M53’, did not exhibit the resistance marker allele despite being resistant to *Foc* STR4 [19] (Table 1). These findings indicate a 62.5% detection rate of resistant genotypes using the marker, suggesting that while the marker is a useful tool for identifying resistance, it may not capture all resistant genotypes due to linkage disequilibrium and resistance sources controlled by other unlinked loci.

Improved diploids are developed by crossing wild diploids with the objective of obtaining hybrids with important agronomical characteristics such as disease resistance. There are three types of improved diploids: (1) first generation − wild diploid × wild diploid, (2) second generation − Improved diploid × wild diploid, and (3) third generation − improved diploid × improved diploid. The third generation may have many wild diploids in its genealogy, whereas the hybrid combines important agronomical characteristics that were distributed among the wild diploids [14].

The screening results suggest that the marker did not detect the resistant allele in diploids including ‘Calcutta 4’, ‘Pisang Lilin’, ‘Tuu Gia’, and ‘Borneo’ despite having good or intermediate levels of resistance/tolerance to *Foc* race 1 and *Foc* TR4 [11,21]. The negative results in marker screening highlight the limitations of this marker in detecting resistance traits at loci other than the target or in genotypes lacking *Musa acuminata* ssp. *malaccensis* origin. This suggests that additional markers may be needed to identify resistance in a broader range of banana genotypes and as well in *Musa acuminata* ssp. *malaccensis*-derived bananas such as ‘Pisang Lilin’. These genotypes are scheduled for field evaluation to assess their resistance to *Foc* TR4, adhering to quarantine protocols in Australia and Colombia. Additionally, we plan to use the putative *Foc* TR4-resistant improved diploids in breeding efforts aimed at introducing sources of *Foc* TR4-resistance into dessert bananas including ‘Prata’, ‘Silk’, and ‘Cavendish’.

This work highlights the importance of improved diploid genotypes, particularly ‘CNPMF 512’ and ‘CNPMF 1323’, which carry resistance to other pests and diseases. These genotypes also possess desirable agronomic traits, including short plant height, early flowering time, and good tillering ability [22]. The genotypes will be field-tested for resistance to *Foc* TR4 in Australia and Colombia, in collaboration with the Department of Agriculture, Fisheries, and Forestry and Agrosavia, to further validate the marker.

This study represents the first report of screening an SNP marker across such an extensive number of banana accessions in Latin America. Given that *Foc* TR4 is already present in Latin America, the urgent need for validation of this SNP marker in genotypes is underscored by its potential impact. Our findings provide valuable insights to expedite resistance breeding in other banana programs worldwide, such as those conducted by IITA for cooking and plantain bananas, and by CIRAD [7,23,24,25].

## Figures and Tables

**Table 1:** Sample number, accession name and number (Embrapa/ITC), type, genealogy, BcoDI digest, and allele nucleotide of 123 Embrapa accessions genotyped with SNP markers *29730-A-SNP1-F2* and *29730-A-SNP1-R2*.

Sample Number	Accession Name	Accession Number	Type	Pedigree	Marker-Allele Defined by BcoDI Digest	SNP and Allele Zygosity	Predicted *Foc* TR4 Responses
**1**	042023-03	042023-03	Improved diploid (AA)	**M53** *×* Cultivar sem nome N^º^ 2	B	C/C	Sus
**2**	091087-02	091087-02	Improved diploid (AA)	**001016–01** (**Borneo** *×* Guyod) *×* 003038–01 (**Calcutta 4** *×* Heva)	B	C/C	Sus
**3**	SH3362^R^	-	Improved diploid (AA)	-	H	T/T	Res
**4**	003004-02	003004-02	Improved diploid (AA)	**Calcutta 4** *×* **Madang**	B	C/C	Sus
**5**	013019-01^R^	013019-01	Improved diploid (AA)	**Malaccensis** *×* **Tjau Lagada**	H	T/C	Res
**6**	041054-04	041054-04	Improved diploid (AA)	003004-01 (**Calcutta 4** *×* Madang) *×* 001004-01 (**Borneo** *×* **Madang**)	B	-	Sus
**7**	050012-02^HR^	050012-02	Improved diploid (AA)	M61 *×* Lidi	B	-	Sus
**8**	086094-15	086094-15	Improved diploid (AA)	03037-02 (**Calcutta 4** *×* Galeo) *×* **SH3263**	H	T/C	Res
**9**	042015-02	042015-02	Improved diploid (AA)	**M53** *×* Madu	B	C/C	Sus
**10**	SH3263^R^	-	Improved diploid (AA)	-	A	T/T	Res
**11**	042085-02	042085-02	Improved diploid (AA)	**M53** *×* 015003-01 (Madu *×* **Calcutta 4**)	B	-	Sus
**12**	042079-06	042079-06	Improved diploid (AA)	**M53** *×* 028003-01 (**Tuu Gia** *×* **Calcutta 4**)	B	C/C	Sus
**13**	017041-01	017041-01	Improved diploid (AA)	**Jari Buaya** *×* 003004–01 (**Calcutta 4** *×* **Madang**)	B	C/C	Sus
**14**	042052-03	042052-03	Improved diploid (AA)	**M53** *×* Kumburgh	B	-	Sus
**15**	042049-04	042049-04	Improved diploid (AA)	**M53** *×* M48	B	C/C	Sus
**16**	003023-03	003023-03	Improved diploid (AA)	**Calcutta 4** *×* S/Nº 2	B	T/C	Res
**17**	086094-20	086094-20	Improved diploid (AA)	03037-02 (**Calcutta 4** *×* Galeo) *×* **SH3263**	H	C/C	Sus
**18**	001016-01^HR^	001016-01	Improved diploid (AA)	**Borneo** *×* Guyod	H	C/C	Sus
**19**	091094-04	091094-04	Improved diploid (AA)	**001016–01** (Borneo *×* Guyod) *×* **SH3263**	B	C/C	Sus
**20**	TH-0301	TH-0301	Improved diploid (AA)	Terrinha *×* **Calcutta 4**	B	-	Sus
**21**	041054-08	041054-08	Improved diploid (AA)	003004-01 (**Calcutta 4** *×* **Madang**) *×* 001004-01 (**Borneo** *×* **Madang**)	B	-	Sus
**22**	058054-03	058054-03	Improved diploid (AA)	003005–01 (**Calcutta 4** *×* **Pahang**) *×* 001004–01 (**Borneo** *×* **Madang**)	B	-	Sus
**23**	089087-01	089087-01	Improved diploid (AA)	013018-01 (**Malaccensis** *×* Sinwobogi) *×* 003038-01 (**Calcutta 4** *×* Heva)	B	-	Sus
**24**	003037-02	003037-02	Improved diploid (AA)	**Calcutta 4** *×* Galeo	B	C/C	Sus
**25**	086079-09	086079-09	Improved diploid (AA)	003037–02 (**Calcutta 4** *×* Galeo) *×* 028003–01 (**Tuu Gia** *×* **Calcutta 4**)	B	-	Sus
**26**	073041-01	073041-01	Improved diploid (AA)	Khai *×* 003004–01 (**Calcutta 4** *×* **Madang**)	B	-	Sus
**27**	013004-04	013004-04	Improved diploid (AA)	**Malaccensis** *×* **Madang**	H	C/C	Sus
**28**	013018-01	013018-01	Improved diploid (AA)	**Malaccensis** *×* Sinwobogi	B	-	Sus
**29**	028003-01	028003-01	Improved diploid (AA)	**Tuu Gia** *×* **Calcutta 4**	B	-	Sus
**30**	091079-03	091079-03	Improved diploid (AA)	01016-01 (**Borneo** *×* Guyod) *×* 028003 (**Tuu Gia** *×* **Calcutta 4**)	B	C/C	Sus
**31**	Tropical	-	Silk hybrid (AAAB)	Yangambi no.2 × **M53**	B	-	Sus
**32**	Belluna	-	Cultivar (AAA)	-	B	-	Sus
**33**	Caipira	-	Cultivar (AAA)	-	H	T/C	Res
**34**	Prata Graúda	-	Prata hybrid (AAB)	SH3642	H	T/C	Res
**35**	Thap-Maeo	ITC1301	Cultivar (AAB)		B	-	Sus
**36**	Prata-Anã	ITC0962	Cultivar (AAB)		B	-	Sus
**37**	BRS Vitória	-	Prata hybrid (AAAB)	Pacovan × **M53**	B	-	Sus
**38**	BRS Preciosa	-	Prata hybrid (AAAB)	Pacovan × **M53**	B	C/C	Sus
**39**	BRS Japira	-	Prata hybrid (AAAB)	Pacovan × **M53**	B	-	Sus
**40**	BRS Garantida	-	Prata hybrid (AAAB)	Pacovan × **M53**	H	C/C	Sus
**41**	BRS Pacovan-Ken	-	Prata hybrid (AAAB)	Pacovan × **M53**	B	-	Sus
**42**	Pacovan	-	Prata Type (AAB)	Prata Type triploid	B	-	Sus
**43**	Platina	ITC0262	Prata hybrid (AAAB)	Prata Anã × **M53**	B	-	Sus
**44**	BRS Caprichosa	-	Prata hybrid (AAAB)	Prata Comum × **M53**	B	-	Sus
**45**	BRS Pioneira	-	Prata hybrid (AAAB)	Prata São Tomé × **M53**	B	-	Sus
**46**	BRS Princesa	-	Silk Hybrid (AAAB)	Yangambi no.2 × **M53**	B	-	Sus
**47**	Pelipita	ITC0095	Cultivar (ABB)	-	B	-	Sus
**48**	Mongolo	-	Plantain (AAB)	-	B	-	Sus
**49**	Red Yade	ITC1140	Plantain (AAB)	-	B	-	Sus
**50**	Comprida	-	Plantain (AAB)	-	B	-	Sus
**51**	Tipo Velhaca	-	Plantain (AAB)	-	B	-	Sus
**52**	Terra Ponta Aparada	-	Plantain (AAB)	-	B	-	Sus
**53**	Pinha	-	Plantain (AAB)	-	B	-	Sus
**54**	Samura B	-	Plantain (AAB)	-	B	C/C	Sus
**55**	Trois Vert	ITC1127	Plantain (AAB)	-	B	-	Sus
**56**	Terra Sem Nome	-	Plantain (AAB)	-	B	-	Sus
**57**	FHIA 21	ITC1306	Hybrid (AAAB)	-	B	C/C	Sus
**58**	Terrinha	-	Plantain (AAB)	-	B	C/C	Sus
**59**	Njock Kon	ITC1133	Plantain (AAB)	-	B	-	Sus
**60**	Curare Enano	ITC0559	Plantain (AAB)	-	B	C/C	Sus
**61**	Chifre De Vaca	-	Plantain (AAB)	-	B	-	Sus
**62**	Terra Maranhão	-	Plantain (AAB)	-	B	-	Sus
**63**	D’Angola	-	Plantain (AAB)	-	B	-	Sus
**64**	CNPMF 0557^R^	111090-07	Improved diploid (AA)	[(M61 × **‘Pisang Lilin’**)] × [(**Malaccensis** × **Tjau Lagada**)]	H	-	Res
**65**	CNPMF 0496^R^	111040-03	Improved diploid (AA)	[(M61 × **‘Pisang Lilin’**)] × [(Terrinha × **Calcutta 4**)]	H	-	Res
**66**	CNPMF 0513^R^	111102-01	Improved diploid (AA)	[(M61 × **‘Pisang Lilin’**)] × [(**M53** × Kumburgh)	B	-	Sus
**67**	CNPMF 0519	116116-01	Improved diploid (AA)	Self-fertilization (wild diploid Tambi)	B	-	Sus
**68**	CNPMF 0536^HR^	9041090-02	Improved diploid (AA)	[(**Calcutta 4** × **Madang**)] × [(**Borneo** × Guyod)]	H	T/C	Res
**69**	CNPMF 0534	041090-20	Improved diploid (AA)	[(**Calcutta 4** × **Madang**)] × [(**Borneo** × Guyod)]	B	C/C	Sus
**70**	CNPMF 0542^R^	094089-01	Improved diploid (AA)	[(**SH3263**)] × [(**Malaccensis** × Sinwobogi)]	H	T/C	Res
**71**	CNPMF 0565	096077-01	Improved diploid (AA)	[(**Calcutta 4** × **Pahang**) × (**Borneo** × **Madang**)] × Khae	B	C/C	Sus
**72**	CNPMF 0572	106090-01	Improved diploid (AA)	[(Khai × (**Calcutta 4** × **Madang**)] × [(**Calcutta 4** × **Madang**)]	B	C/C	Sus
**73**	CNPMF 0612	098094-01	Improved diploid (AA)	[(**M53** × Madu) × Madu)] × **SH3263**	H	C/C	Sus
**74**	CNPMF 0731^R^	088079	Improved diploid (AA)	[(**Malaccensis** × **Madang**)] × [(**Tuu Gia** × **Calcutta 4**)]	H	T/C	Res
**75**	CNPMF 0767	088100	Improved diploid (AA)	[(**Malaccensis** × **Madang**)] × [(Khai × (**Calcutta 4** × **Madang**)]	B	C/C	Sus
**76**	CNPMF 0811	106096	Improved diploid (AA)	[(Khai × (**Calcutta 4** × **Madang**)] × [(**Calcutta 4** × **Pahang**) × (**Borneo** × **Madang**)]	B	-	Sus
**77**	CNPMF 0037	098090-01	Improved diploid (AA)	[(**M53** × Madu)] × [(**Malaccensis** × **Tjau Lagada**)]	B	-	Sus
**78**	CNPMF 0898	PA × 119	Improved diploid (AA)	[(Prata Anã)] × [(**Malaccensis** × Sinwobogi) × (**Calcutta 4** × Galeo)]	B	-	Sus
**79**	CNPMF 0038	098090-01	Improved diploid (AA)	[(**M53** × Madu)] × [(**Malaccensis** × **Tjau Lagada**)]	B	-	Sus
**80**	CNPMF 1102	093117	Improved diploid (AA)	[(**Jari Buaya** × (**Calcutta 4** × **Madang**)] × [(**Borneo** × Guyod) × (**Tuu Gia** × **Calcutta 4**)]	B	-	Sus
**81**	CNPMF 1171	088108	Improved diploid (AA)	[(**Malaccensis** × **Madang**)] × [(**M53** × (**Tuu Gia** × **Calcutta 4**)]	B	-	Sus
**82**	CNPMF 0993^R^	117100	Improved diploid (AA)	[(**Borneo** × Guyod) × (**Tuu Gia** × **Calcutta 4**)] × [(Khai × (**Calcutta 4** × **Madang**)]	B	-	Sus
**83**	CNPMF 1323	089087	Improved diploid (AA)	[(**Malaccensis** × Sinwobogi)] × [(**Calcutta 4** × Heva)]	B	-	Sus
**84**	CNPMF 1105	123097	Improved diploid (AA)	[(**Borneo** × Guyod) × (**Calcutta 4** × Heva)] × [(**Calcutta 4** × **Madang**)]	B	-	Sus
**85**	CNPMF 0998^R^	091124	Improved diploid (AA)	[(**Borneo** × Guyod)] × [(**Borneo** × Guyod) × **SH3263**]	B	-	Sus
**86**	CNPMF 0978	041040	Improved diploid (AA)	[(**Calcutta 4** × **Madang**)] × [(Terrinha × **Calcutta 4**)]	B	C/C	Sus
**87**	CNPMF 1272	123079	Improved diploid (AA)	[(**Borneo** × Guyod) × (**Calcutta 4** × Heva)] × [(**Tuu gia** × **Calcutta 4**)]	B	C/C	Sus
**88**	CNPMF 1286	041040	Improved diploid (AA)	[(**Calcutta 4** × **Madang**)] × [(Terrinha × **Calcutta 4**)]	B	C/C	Sus
**89**	M53^R^	-	Landrace cultivar (AA)	**Malaccensis**–Kedah × banksii-Samoa) × (Paka × Banksii-Samoa)	B	C/C	Sus
**90**	Pisang Jaran	ITC0678	Wild diploid (AA)	-	B	C/C	Sus
**91**	Malbut	-	Wild diploid (AA)	-	B	C/C	Sus
**92**	Calcutta4	ITC0249	Wild diploid (AA)	-	B	C/C	Sus
**93**	Birmanie	-	Wild diploid (AA)	-	B	C/C	Sus
**94**	Akondro Mainty	ITC0281	Mchare landrace (AA)	-	B	-	Sus
**95**	NBA-14	ITC0267	Wild diploid (AA)	-	B	C/C	Sus
**96**	Khai Nai On	ITC0663	Wild diploid (AA)	-	B	C/C	Sus
**97**	Khai	ITC0532	Wild diploid (AA)	-	B	C/C	Sus
**98**	Pisang Berlin	ITC0611	Edible diploid (AA)	-	B	T/C	Res
**99**	Khi Maeo	-	Wild diploid (AA)	-	B	-	Sus
**100**	Borneo	ITC0253	Wild diploid (AA)	-	B	C/C	Sus
**101**	Mambee Thu	ITC0612	Edible diploid (AA)	-	B	C/C	Sus
**102**	Niyarma Yik	ITC0269	Edible diploid (AA)	-	B	C/C	Sus
**103**	*M. a.* spp. *malaccensis*	ITC0399	Wild diploid(AA)	-	H	-	Res
**104**	Pisang Tongat	ITC0063	Edible diploid (AA)	-	B	C/C	Sus
**105**	Pa Mysore2	ITC0668	Wild diploid (AA)	-	H	C/C	Sus
**106**	Pisang Jari Buaya	ITC0690	Edible diploid (AA)	-	B	C/C	Sus
**107**	Tuu Gia	ITC0610	Edible diploid (AA)	-	B	C/C	Sus
**108**	Pisang Nangka	ITC0004	Edible triploid (AAB)	-	B	C/C	Sus
**109**	Tjau Lagada	ITC0090	Wild diploid (AA)	-	B	C/C	Sus
**110**	Tong Dok Mak	ITC0411	Wild diploid (AA)	-	H	C/C	Sus
**111**	M-61	-	Improved diploid (AA)		H	C/C	Sus
**112**	Pisang Mas	ITC1403	Edible diploid (AA)	-	H	T/C	Res
**113**	Pisang Cici	ITC0681	Wild diploid (AA)	-	B	C/C	Sus
**114**	Saney	-	Wild diploid (AA)	-	B	C/C	Sus
**115**	Yangambi no.2	ITC1275	Cultivar (AAB)	-	B	C/C	Sus
**116**	Yangambi Km5	ITC1123	Cultivar (AAB)	-	H	T/C	Res
**117**	Highgate	ITC0263	Cultivar (AAA)	-	B	-	Sus
**118**	BRS Pacoua PV 03-76	-	Prata hybrid (AAAB)	Pacovan × **Calcutta 4**	B	C/C	Sus
**119**	PA 42-38	PA 42-38	Prata hybrid (AAAB)	Prata Anã × **M53**	B	C/C	Sus
**120**	PA 42-28	PA 42-28	Prata hybrid (AAAB)	Prata Anã × **M53**	B	C/C	Sus
**121**	PA 42-19	PA 42-19	Prata hybrid (AAAB)	Prata Anã × **M53**	-	C/C	Sus
**122**	M48	-	Improved diploid (AA)	-	B	C/C	Sus
**123**	Ouro	-	Cultivar (AA)	-	H	T/T	Res

B = Marker homozygous for the susceptible allele, predicted to be *Foc* TR4-susceptible: H = Marker heterozygous, predicted to be *Foc* TR4-resistant; A = Marker homozygous for the resistant allele, predicted to be *Foc* TR4-resistant; Nucleotide ‘T’ corresponds to the resistant allele and nucleotide ‘C’ the susceptible allele; ‘T/C’ and ‘T/T’ are predicted to be resistant whereas ‘C/C’ = is predicted to be susceptible. “HR’ and “R” within the “Accession name” column indicate accessions that were “Highly Resistant” and “Resistant”, respectively, to *Foc* STR4, as determined in a previous study [19]. Accessions listed in bold within the “Pedigree” column denote parental accessions previously shown to be resistant to *Foc* TR4. Sanger sequencing was used to determine the SNP identity at this locus. “-” within the “SNP and allele zygosity” column indicates that Sanger sequencing was not performed for these particular accessions.

## Data Availability

The authors will provide data upon request.

## Supplementary Materials

The following supporting information can be downloaded at https://www.mdpi.com/article/10.3390/jof10120839/s1: Supplementary Figure S1: Sequencing chromatograms for the *Macma4_03_g32560* gene were generated using both forward and reverse primers.
