# Supplementary material for: Toward Marker-Assisted Selection in Breeding for Fusarium Wilt Tropical Race-4 Type Resistant Bananas
Source: J Fungi (Basel). 2024 Dec 4;10(12):839. doi: 10.3390/jof10120839 (PMC11676971; doi:10.3390/jof10120839)
Supplement: Supplementary file 1 [file jof-10-00839-s001.zip › jof-3300690-supplementary.pdf]

Toward Marker-Assisted Selection in Breeding for Fusarium wilt Tropical Race-4 Type Resistant Bananas

Claudia Fortes Ferreira [1,*,†], Andrew Chen [2,†], Elizabeth A. B. Aitken [2], Rony Swennen [3,4], Brigitte Uwimana [3], Anelita de Jesus Rocha [5], Julianna Matos da Silva Soares [5], Andresa Priscila de Souza Ramos[1] and Edson Perito Amorim [1]

Supplemental Material

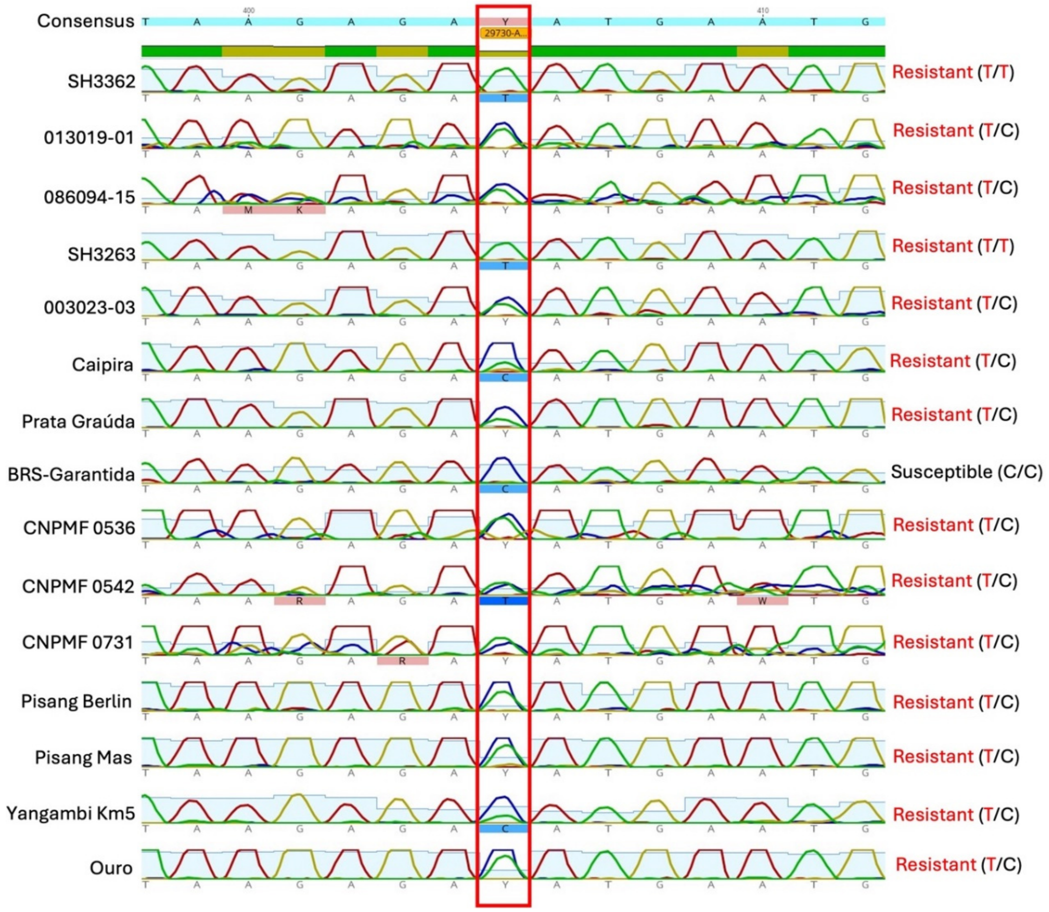

Figure S1. Sequencing chromatograms for the *Macma4_03_g32560* gene were generated using both forward and reverse primers. These sequences were then aligned using the MAFFT alignment tool in Geneious Prime to confirm the presence of the target SNP. Within the marker sequences, the informative SNP position interrogated across the genotypes is highlighted in red.
